# Skin Grafting in Pyoderma Gangrenosum

**Published:** 2018-05-18

**Authors:** Marco Romanelli, Agata Janowska, Teresa Oranges, Valentina Dini

**Affiliations:** Department of Dermatology, University of Pisa, Pisa, Italy

**Keywords:** pyoderma gangrenosum, allograft, pathergy, adalimumab, chronic wounds

## DESCRIPTION

**Figure 1:**
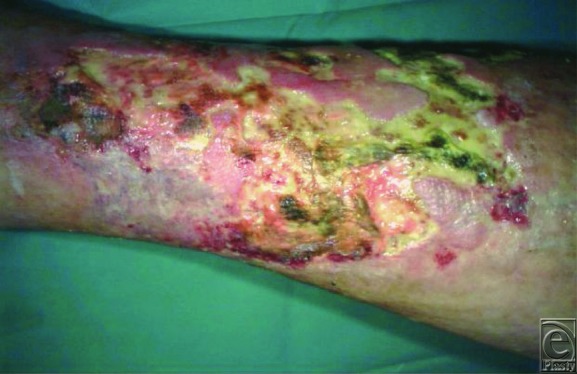
Wound bed status of pyoderma gangrenosum at baseline.

**Figure 2:**
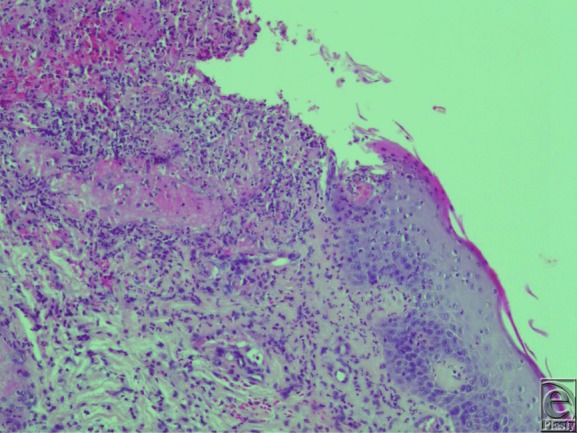
Tissue biopsy of pyoderma gangrenosum showing aspecific dermal neutrophilic infiltrate.

A 75-year-old woman presented with multiple painful necrotic ulcers of the lower leg lasting since 2 months (Fig 1). Doppler studies of the venous and arterial systems were within normal limits. The diagnosis of pyoderma gangrenosum (PG) was based on clinical and histopathological presentation showing a dermal aspecific neutrophilic infiltrate (Fig 2).

**Figure 3:**
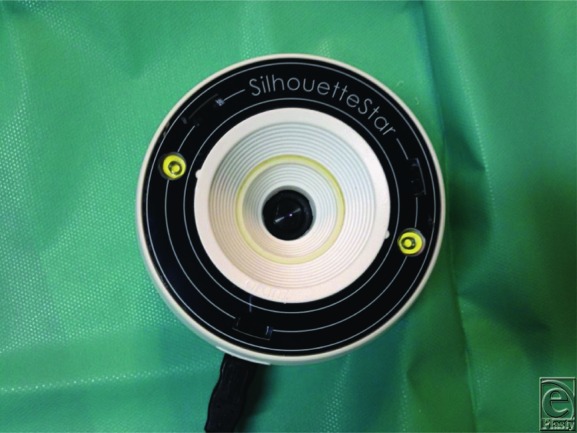
Silhouette Star camera for 3D wound imaging.

Current blood screening with complete blood cell count, renal and liver function tests, lipid profile, the serum electrolytes, serum electrophoresis, clinical antibody profile, serology for rheumatoid factor, thyroid function test, and blood sugar evaluation was within normal limits. The patient had a history of pathergy after antibiotic injection. Comorbidities were hypertension, atrial fibrillation, and rheumatoid arthritis in remission. No history of allergies was reported. A digital camera and a 3-dimensional measurement, imaging, and documentation system (Star Aranz, Aranz Medical, New Zealand) (Fig 3) were used to take the photographs and to provide the precise measurement of size and healing trend development. The patient was followed up until the complete resolution of the disease. Pyoderma gangrenosum was managed with 0.5 mg/kg of oral methylprednisolone, with local wound care, based on the principles of the wound bed preparation^1^ and inelastic bandage therapy.^2^ For the first 3 weeks, the local treatment was characterized by autolytic debridement using hydrogel (NU-GEL, Systagenix, United Kingdom), binding bacteria dressings (Cutimed Sorbact, BSN, Germany) 3 times per week to reduce necrosis and fibrin. The wound care was continued with hydrofiber (Aquacel, Aquacel, ConvaTec, New Jersey) and inelastic bandage therapy twice per week for 2 weeks. Because of the worsening hypertension, the oral corticosteroid therapy was tapered and then interrupted. We did not use cyclosporine because of the hypertension, and we performed anti-TNFα therapy (adalimumab) 40 mg weekly.^3^ After 1 month of using adalimumab, hydrofiber (Aquacel) and inelastic bandage, the ulcers healed with more granulation tissue, along with a reduction in the inflammation, and the patient reported a reduction in pain level. The ulcers were characterized by superficial granulation tissue, absence of infection, critical colonization, or inflammation.

**Figure 4:**
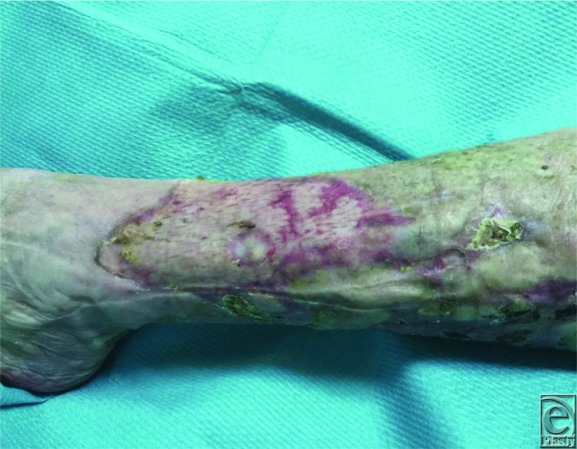
Wound bed at 3 weeks after skin grafting and systemic therapy with anti TNF alfa drug.

We decided to perform an allograft due to a pathergy history and to the wound bed clinical aspects. The allograft came from a cadaver skin and was cryopreserved at −80°C. The ulcers were cleaned with saline solution and antiseptic solution. The allograft was meshed 3:1 and fixed on the wounds with Steri-Strip skin closure and then covered with binding bacteria dressing and inelastic bandage. We provided the procedure on the same day as a “day surgery” treatment. The follow-up was performed twice a week for 3 weeks. We observed a perfect engraftment of the allograft during the final visit (Fig 4).

## QUESTIONS

1. What is pyoderma gangrenosum?
2. How is it diagnosed?
3. What is pathergy?
4. How is it treated?

## DISCUSSION

Pyoderma gangrenosum is a rare, painful neutrophilic dermatosis that involves the skin and other organs. The diagnosis is based on the presence of suggestive clinical and histological aspects and the exclusion of other conditions. Typical histological evaluation shows epidermal ulceration and sterile neutrophilic infiltration without vasculitis or without granuloma formation, but it can be often aspecific.^4^

Pathergy can occur in 30% of patients with PG. Pathergy is a condition in which a minor trauma can cause a development of PG at the site of trauma.^5^ Considering this aspect, it is not recommended to perform surgical debridement in this condition and is mandatory to consider that surgical procedures in any anatomical site may induce the pathergy phenomenon.

Pyoderma gangrenosum is extremely difficult to treat, and it often needs the association of systemic treatments combined with a correct wound bed preparation approach.^6^

Corticosteroids are considered the first-line therapy for severe disease, and cyclosporine is a good second-line option in patients with no hypertension or renal impairment. Other options include other immunosuppressive drugs, intravenous immunoglobulin, and targeted therapies such as anti-TNFα, anti-interleukin 1, anti-interleukin 12,23.^5^^,^^7^^,^^8^

A noninvasive procedure such as allograft was preferred because of the pathergy history of the patient. Other surgical approaches have also been described in PG, such as the use of negative pressure wound therapy associated with split-thickness skin grafting under adequate immunosuppression.^9^

A combination therapy of anti-TNFα and allograft can improve a graft engraftment with a reduction in immune response against the allograft. In this report, allograft and anti-TNFα (adalimumab) represented an effective association therapy for hard-to-heal PG.
